# Association Between Frailty, Locomotive Syndrome, and Subjective Well-Being Among Community-Dwelling 80-Year-Old Adults in Japan: A Pilot Study

**DOI:** 10.3390/healthcare14091254

**Published:** 2026-05-06

**Authors:** Tamaki Hirose, Yohei Sawaya, Masahiro Ishizaka, Naori Hashimoto, Tomohiko Urano

**Affiliations:** 1 Department of Physical Therapy, School of Health Sciences, International University of Health and Welfare, 2600-1 Kitakanemaru, Otawara 324-8501, Tochigi, Japan; n-tamaki@ihwg.jp (T.H.); sawaya@ihwg.jp (Y.S.); ishizaka@ihwg.jp (M.I.); 2 Senior Services Division of Otawara, 1-4-1 Honcho, Otawara 324-8641, Tochigi, Japan; 3 Department of Geriatric Medicine, School of Medicine, International University of Health and Welfare, 4-3 Kozunomori, Narita 286-8686, Chiba, Japan

**Keywords:** frailty, locomotive syndrome, subjective well-being, life satisfaction, happiness

## Abstract

**Objectives:** Despite growing interest in locomotive syndrome (LS), its relationship with subjective well-being remains unclear, highlighting a gap in the existing literature. Therefore, as a pilot study providing preliminary insights, this study aimed to clarify the association of subjective well-being (life satisfaction and happiness) with LS and frailty, a key concept in geriatric medicine, among community-dwelling individuals. **Methods:** We conducted a cross-sectional study of 111 older adults aged 80 years living in City A, Tochigi Prefecture, Japan. LS (5-question Geriatric Locomotive Function Scale [GLFS-5]), frailty (Kihon Checklist [KCL]), and subjective well-being (life satisfaction and happiness) were assessed. Group comparisons were performed based on life satisfaction (Satisfied vs. Unsatisfied) and happiness (Happy vs. Unhappy). Binary logistic regression analyses were also conducted, with LS and frailty as dependent variables and subjective well-being (Satisfied–Happy [reference], Satisfied–Unhappy, Unsatisfied–Unhappy) as independent variables. **Results:** LS was observed in 32 participants (28.8%), and frailty was observed in 25 participants (22.5%). In the group comparisons, the prevalence of LS and frailty was significantly higher in the Unsatisfied and Unhappy groups. In multivariable analyses, the Unsatisfied–Unhappy group was significantly associated with LS (*p* = 0.002) and frailty (*p* = 0.031). **Conclusions:** Among community-dwelling individuals aged 80 years, low life satisfaction and happiness were shown to be associated with LS and frailty.

## 1. Introduction

In 2022, the Japanese Medical Science Federation issued the “Declaration of the Medical Society for Overcoming Frailty and Locomotive Syndrome,” which positioned countermeasures against frailty and locomotive syndrome (LS) as key priorities for extending healthy life expectancy [1]. LS is defined as a condition in which mobility declines owing to impairments of the musculoskeletal system, including the bones, joints, muscles, and intervertebral discs. As the condition progresses, walking ability and activities of daily living become restricted, leading to an increased risk of requiring long-term care [2,3,4]. In contrast, frailty refers to a state of heightened vulnerability due to age-related declines in physiological reserve, encompassing not only physical but also psychological and social aspects [5]. Frailty has been linked to various negative health outcomes, such as falls, disability in daily functioning, hospitalization, and increased risk of mortality [6,7,8]. Although LS and frailty are important conditions that contribute to poorer healthy life expectancy, they represent different clinical constructs. LS primarily reflects a decline in mobility, whereas frailty reflects multidimensional vulnerability.

In recent years, subjective well-being has attracted increasing attention as an indicator of the comprehensive assessment of the health of older adults. Subjective well-being is a concept composed of a cognitive component that reflects how individuals evaluate their own lives and an affective component, including feelings of happiness. It is an important psychosocial indicator of healthy longevity and successful ageing [9]. Higher levels of subjective well-being have been associated with better health maintenance and a lower risk of mortality [10,11,12]. Recent studies of community-dwelling individuals in Japan have shown that life satisfaction and happiness are associated with factors such as functional ability and sleep satisfaction [13,14].

Accumulating evidence has clarified the association between frailty and subjective well-being. A 2024 study of middle-aged and older Japanese persons has reported that even in the presence of strong social isolation, individuals with higher levels of subjective well-being had a lower risk of physical frailty [15]. Furthermore, a study published in 2025 has reported that lower subjective well-being was associated with deterioration of frailty and mortality [16]. These findings suggest that subjective well-being is not merely a psychological indicator but is also closely related to physical vulnerability in older adults.

In contrast, most previous studies examining factors associated with LS have focused on physical aspects such as walking ability, physical performance, nutritional status, and lifestyle factors [17,18,19,20], and no studies have investigated the association between LS and subjective well-being. Furthermore, no study has examined frailty and LS in the same population or compared their associations with subjective well-being. Moreover, even within the concept of subjective well-being, it has been suggested that factors associated with cognitive evaluations, such as life satisfaction, and affective evaluations, such as happiness, do not necessarily coincide [11,12]. Therefore, simultaneously assessing life satisfaction and happiness as representative indicators of subjective well-being and examining their associations with LS and frailty may provide a more comprehensive understanding of the relationship between physical decline and psychosocial health among older adults.

In this context, as a pilot study providing preliminary insights, the present study investigated how LS and frailty are related to subjective well-being, evaluated using life satisfaction and happiness, in community-dwelling older adults.

## 2. Materials and Methods

### 2.1. Study Design

A cross-sectional survey was conducted in July 2025. Ethical approval for the study was obtained from the Ethics Committee of the International University of Health and Welfare (approval numbers: 22-Io-25). Informed consent was obtained using an opt-out approach via the official website of City A in Tochigi Prefecture. The study complied with the principles outlined in the Declaration of Helsinki.

### 2.2. Study Setting and Participants

**Figure 1:**
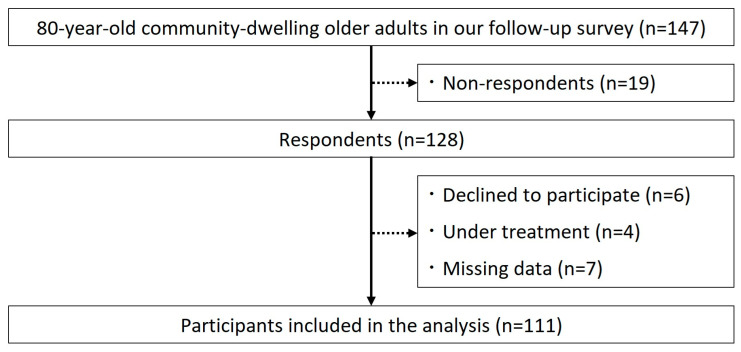
Flow diagram of participants.

The participants comprised a community-dwelling single-year cohort of 147 80-year-old adults residing in City A, Tochigi Prefecture, Japan. This study was conducted using a mail-based questionnaire as part of an ongoing follow-up study [21,22,23]. All participants responded to the follow-up survey and were not certified as requiring long-term care. Of the 147 individuals, 128 responded to the survey. Participants who declined to participate (n = 6), were unable to cooperate with the survey due to ongoing medical treatment (n = 4), or were missing responses to questionnaire items (n = 7) were excluded. Consequently, 111 participants (61 males and 50 females) were included in the final analysis (Figure 1).

### 2.3. Frailty

The Kihon Checklist (KCL), a 25-item questionnaire encompassing seven domains, was used to assess frailty [24]. Each item evaluates the presence or absence of functional impairment in daily life and is scored as 0 (no impairment) or 1 (impairment). The total score of the 25 items (range: 0–25) reflects cumulative functional decline, with higher scores indicating greater deterioration in functional status. Based on established criteria from previous studies, participants were grouped into robust (0–3 points), pre-frailty (4–7 points), and frailty (≥8 points) using the total KCL score [24,25].

### 2.4. Locomotive Syndrome

LS was assessed using the 5-question Geriatric Locomotive Function Scale (GLFS-5). The GLFS-5 is composed of five questions assessing locomotive function, with each item scored on a scale of 0 to 4; higher scores indicate more severe mobility decline. Based on the established criterion that a total GLFS-5 score of ≥6 indicates the presence of LS [26], the number of participants meeting this criterion was calculated in this study. Additionally, the total GLFS-5 and individual item scores were used as evaluation indicators.

### 2.5. Subjective Well-Being and Group Classification

Subjective well-being was assessed using questionnaire items on life satisfaction and happiness, referring to the participants’ status over the past month. Life satisfaction was assessed using the question “Are you satisfied with your daily life?”, with four response options on a Likert scale: “Satisfied,” “Moderately satisfied,” “Moderately dissatisfied,” and “Dissatisfied.” Participants who answered “Satisfied” or “Moderately satisfied” were classified into the satisfied group, whereas those who answered “Moderately dissatisfied” or “Dissatisfied” were classified into the unsatisfied group [27,28]. Happiness was assessed using the question “How happy do you feel at present?” The responses were rated on an 11-point scale ranging from 0 (“Very unhappy”) to 10 (“Very happy”). In this study, participants with a score of ≥7 were defined as the happy group, whereas those with a score of ≤6 were defined as the unhappy group, in accordance with previous studies [29,30].

Based on these results, participants were classified into three groups:

- Satisfied–Happy: participants classified as both satisfied and happy.
- Satisfied–Unhappy: participants classified as satisfied and unhappy.
- Unsatisfied–Unhappy: participants classified as unsatisfied and unhappy.

Among the 111 participants included in this study, none were classified as Unsatisfied–Happy. These group classifications were conducted with reference to the grouping methods used in previous studies [22,23,31].

### 2.6. Other Variables

Data were collected regarding basic characteristics, such as sex, height, body weight, body mass index (BMI), living alone, hobbies, participation in community activities, and medical conditions (hypertension, hyperlipidemia, and cancer).

### 2.7. Statistical Analysis

For exploratory analysis, group comparisons were conducted based on life satisfaction (satisfied vs. unsatisfied) and happiness (happy vs. unhappy). The variables compared between the groups included height, body weight, BMI, living alone, presence of hobbies, participation in community activities, medical history (hypertension, hyperlipidemia, and cancer), prevalence of LS, total score and individual items of GLFS-5, prevalence of frailty, and total score and seven domain scores of KCL. For group comparisons, the chi-square test or Fisher’s exact test was used for categorical variables, the unpaired t-test for continuous variables, and the Mann–Whitney U test for score variables. Additionally, comparisons among the three groups (Satisfied–Happy, Satisfied–Unhappy, and Unsatisfied–Unhappy) were performed using the chi-square or Fisher’s exact tests, one-way analysis of variance, and the Kruskal–Wallis test, with multiple comparisons conducted following the latter two.

Furthermore, binary logistic regression analyses were performed with the presence of LS and frailty as dependent variables and subjective well-being (three-group classification based on the combination of life satisfaction and happiness) as the independent variable to examine the associations of LS and frailty with subjective well-being. Regarding the independent variable, “Satisfied–Happy” was set as the reference category. No covariates were included in the crude model, whereas Model I was adjusted for sex and BMI, considering the limited number of covariates relative to the sample size [32] and their established associations with LS and frailty [33,34,35,36]. All analyses were carried out using SPSS version 25 (IBM Japan, Tokyo, Japan), and statistical significance was defined as a *p*-value < 0.05.

## 3. Results

The characteristics of all enrolled participants are presented in Table 1. Among the 111 participants included in this study, 32 (28.8%) were classified as having LS and 25 (22.5%) as having frailty.

In the comparison between the satisfied and unsatisfied groups based on life satisfaction, significant differences were observed in the prevalence of LS (*p* = 0.002), GLFS-5 items [Q1 (*p* = 0.002), Q2 (*p* = 0.001), Q3 (*p* = 0.007), Q4 (*p* = 0.007), and Q5 (*p* = 0.004)], total GLFS-5 score (*p* = 0.001), prevalence of frailty (*p* = 0.020), KCL domains [physical function (*p* = 0.032), oral function (*p* = 0.007), outdoor activity (*p* = 0.034), depression (*p* < 0.001)], and total KCL score (*p* = 0.002) (Table 2). In the comparison between the happy and unhappy groups based on happiness, statistically significant differences were observed in the prevalence of LS (p = 0.027), GLFS-5 items [Q1 (*p* = 0.022), Q2 (*p* = 0.016), Q4 (*p* = 0.003), and Q5 (*p* = 0.013)], total GLFS-5 score (*p* = 0.012), prevalence of frailty (*p* = 0.011), KCL domains [oral function (*p* = 0.031), outdoor activity (*p* = 0.034), cognitive function (*p* = 0.017), and depression (*p* = 0.001)], and total KCL score (*p* = 0.002) (Table 3). The results of comparisons among the three groups (Satisfied–Happy, Satisfied–Unhappy, and Unsatisfied–Unhappy) are presented in Table 4.

Binary logistic regression analyses were performed to examine the association of LS with life satisfaction and happiness. In both the crude model (OR = 9.3, 95% confidence interval [CI] = 2.2–38.8, *p* = 0.002, accuracy = 75.7%) and Model I (OR = 10.5, 95 % CI = 2.4–45.5, *p* = 0.002, accuracy = 76.4%), being Unsatisfied–Unhappy was significantly associated with LS (Table 5). Similarly, binary logistic regression analyses examining the association between frailty, life satisfaction, and happiness showed that being Unsatisfied–Unhappy was significantly associated with frailty in both the crude model (OR = 4.4, 95% CI = 1.2–16.9, *p* = 0.029, accuracy = 77.5%) and Model I (OR = 4.5, 95% CI = 1.1–17.8, *p* = 0.031, accuracy = 78.2%) (Table 6). All logistic regression models exhibited an acceptable fit based on the Hosmer–Lemeshow test.

## 4. Discussion

In the present pilot study, subjective well-being was evaluated from two perspectives—life satisfaction (cognitive component) and happiness (affective component)—in community-dwelling older adults, and their associations with LS and frailty were examined within the same population. The results showed that the “Unsatisfied–Unhappy” group, characterized by concomitantly low levels of life satisfaction and happiness, was significantly associated with both LS and frailty. These findings suggest that a state in which multiple dimensions of subjective well-being are simultaneously low may be more strongly related to mobility decline and increased vulnerability in older persons. Although previous studies have reported an association between subjective well-being and frailty, to the best of our knowledge, no study has examined the association between subjective well-being and LS [15,16]. Furthermore, no study has simultaneously evaluated LS and frailty within the same population while considering both the cognitive and affective components of subjective well-being. Therefore, given its pilot nature, the findings of this study should be interpreted with caution; however, by comprehensively examining these factors within a single population, it provides novel insights into the relationship between mobility decline and psychosocial health in older adults.

In the exploratory group comparisons in the present study, the group with lower subjective well-being had a higher prevalence of LS and higher GLFS-5 total scores. Significant differences were observed in all GLFS-5 items for life satisfaction and in four items for happiness. GLFS-5 reflects self-perceived mobility-related difficulties, including pain, movement, and challenges in the activities of daily living. The significant differences across multiple items may indicate that older adults with lower subjective well-being perceive broader declines in mobility and greater physical difficulties in daily life. In addition, the group with lower subjective well-being showed a higher prevalence of frailty and higher total KCL scores. Significant differences were observed in several KCL subdomains, including physical function, oral function, going out, and depressive mood. In addition, a similar trend was observed in comparisons among the three groups, suggesting that lower subjective well-being is associated not only with declines in mobility but also with multiple aspects of functional status, such as reduced activity range and changes in psychological conditions. Recent studies have shown that lower subjective well-being is related to adverse health outcomes [16]. Moreover, a study of community-dwelling older adults in Japan has suggested that individuals in their 80s may be more susceptible to influences on subjective well-being than those in their 60s and 70s [14]. Taken together, these findings suggest that LS, characterized by mobility decline, and frailty, reflecting multidimensional functional deterioration, may be closely linked to subjective well-being in older adults.

In this study, life satisfaction and happiness were assessed simultaneously, and multivariate analyses using a classification of subjective well-being showed that the “unsatisfied and unhappy” group was significantly associated with both LS and frailty. These findings form the basis of the main interpretation of the study results. In other words, when both life satisfaction and happiness were low, the association between decline in physical function and impairment in daily functioning tended to be stronger. A distinctive feature of this study is that it focused not only on life satisfaction or happiness individually but also on the state of subjective well-being defined by the combination of both indicators. Life satisfaction is generally regarded as an indicator of cognitive evaluation of one’s overall life, whereas happiness reflects daily affective states. Therefore, the findings of this study suggest that a condition in which multiple dimensions of subjective well-being are simultaneously low may be more strongly associated with physical vulnerability in older adults. These results also highlight the importance of evaluating subjective well-being from multiple perspectives when assessing the health status of older adults.

Recently, increasing attention has been paid not only to disease treatment but also to prevention and health promotion. Conventional approaches for preventing LS and frailty have emphasized the importance of promoting exercise, adequate nutrition, and social participation [21,22,23,37,38,39]. In the present study, lower subjective well-being was associated with both LS and frailty. Subjective well-being is considered a multidimensional construct [10], and support strategies that take into account older adults’ values and life context—namely, their individuality or “personhood”—may be required. While the generalizability of these findings is limited to specific age groups or regions, this study provides a basis for further discussion. The observed trends align with existing evidence regarding the association between subjective well-being and health indicators. These findings suggest that, in addition to physical and social factors such as exercise, nutrition, and social participation, it may be beneficial to consider subjective well-being—including life satisfaction and happiness—as a relevant factor in the context of locomotive syndrome and frailty.

This study had several limitations. First, it employed a cross-sectional design and included a small sample of community-dwelling older adults from a single age group in one city, which may limit the generalizability of the results. Therefore, no participants were classified as Unsatisfied–Happy, and the possibility of selection bias could not be excluded. Furthermore, due to the limited number of events, social and health-related factors could not be included as covariates, and residual confounding may remain. Although the model showed acceptable goodness-of-fit, the precision of the regression estimates may be limited and potentially unstable. Additionally, because of the cross-sectional design of the study, a causal relationship between subjective well-being and LS or frailty could not be determined. Second, this study involved a questionnaire survey in collaboration with the municipal government. Considering the burden on participants, LS was assessed using GLFS-5, which has established reliability and validity, instead of the 25-question Geriatric Locomotive Function Scale (GLFS-25) [26]. Although GLFS-5 is useful as a simple assessment tool, the range of aspects that can be evaluated may be more limited than that of GLFS-25. Additionally, the use of different assessment scales for life satisfaction and happiness, as well as the lack of any detailed information regarding participants’ medical conditions, also represents a significant limitation. Third, subjective well-being and life satisfaction may have been overestimated in their associations due to a conceptual overlap with certain items of the KCL. In addition, these variables, as well as frailty, were all based on self-reported measures, and the potential influence of information bias should therefore be considered. Overall, future studies are warranted to enhance the generalizability of these findings by employing larger sample sizes and longitudinal designs, as well as by conducting multicenter studies that include participants from multiple regions.

## 5. Conclusions

This pilot study examined the associations of subjective well-being with LS and frailty among community-dwelling individuals. The results showed that low life satisfaction and happiness were significantly associated with LS and frailty.

## Figures and Tables

**Table 1:** Characteristics of all participants (n = 111).

Sex	
Male	61, 55.0%
Female	50, 45.0%
Height, cm	158.2 (9.6)
Weight, kg	58.0 (10.4)
BMI, kg/m^2^	23.1 (2.9)
Living alone	9, 8.1%
Engaging in hobbies	56, 50.5%
Community activity participation	51, 45.9%
Hypertension	68, 61.3%
Hyperlipidemia	15, 13.5%
Cancer	8, 7.2%
LS	32, 28.8%
GLFS-5 total score	2.0 [0.0–6.0]
Frailty	
Robust	49, 44.1%
Pre-frailty	37, 33.3%
Frailty	25, 22.5%
KCL total score	4.0 [1.0–7.0]

Data are reported as n, mean (standard deviation) or median [interquartile range]. BMI: body mass index. GLFS-5: The 5-question Geriatric Locomotive Function Scale. KCL: Kihon Checklist. LS: locomotive syndrome.

**Table 2:** Comparison of locomotive syndrome and frailty items between the satisfied and unsatisfied groups.

		Satisfied Group (n = 100)	Unsatisfied Group (n = 11)	*p* Value
Sex, female		46, 46.0%	4, 36.4%	0.751
Height, cm		157.9 (9.6)	160.8 (8.8)	0.343
Weight, kg		58.0 (10.7)	58.0 (7.6)	0.999
BMI, kg/m^2^		23.1 (3.0)	22.4 (1.9)	0.411
Living alone		7, 7.0%	2, 18.2%	0.218
Engaging in hobbies		52, 52.0%	4, 36.4%	0.325
Community activity participation		47, 47.0%	4, 36.4%	0.502
Hypertension		60, 60.0%	8, 72.7%	0.525
Hyperlipidemia		11, 11.0%	4, 36.4%	0.041 *
Cancer		8, 8.0%	0, 0.0%	1.000
LS				
Prevalence		24, 24.0%	8, 72.7%	0.002 *
Q1 Going up and down stairs		0.5 [0.0–1.0]	2.0 [1.0–2.0]	0.002 *
Q2 Walking briskly		1.0 [0.0–1.0]	2.0 [1.0–2.0]	0.001 *
Q3 Keep waking without rest		1.0 [0.0–1.0]	1.0 [1.0–3.0]	0.007 *
Q4 Carrying objects 2 kg		0.0 [0.0–1.0]	1.0 [0.0–1.0]	0.007 *
Q5 Having load-bearing tasks		0.0 [0.0–1.0]	1.0 [1.0–2.0]	0.004 *
GLFS-5 total score		2.0 [0.0–5.0]	7.0 [4.0–9.0]	0.001 *
Frailty				
Status	Robust	48, 48.0%	1, 9.1%	0.020 *
	Pre-frailty	32, 32.0%	5, 45.5%	
	Frailty	20, 20.0%	5, 45.5%	
Domains	Activities of daily living	0.0 [0.0–1.0]	0.0 [0.0–1.0]	0.797
	Physical function	1.0 [0.0–2.0]	2.0 [1.0–3.0]	0.032 *
	Nutrition	0.0 [0.0–1.0]	0.0 [0.0–1.0]	0.789
	Oral function	0.0 [0.0–1.0]	2.0 [0.0–3.0]	0.007 *
	Outdoor activity	0.0 [0.0–0.0]	0.0 [0.0–1.0]	0.034 *
	Cognitive function	0.0 [0.0–1.0]	1.0 [0.0–1.0]	0.051
	Depression	1.0 [0.0–2.0]	4.0 [1.0–5.0]	<0.001 *
KCL total score		4.0 [1.0–6.0]	7.0 [5.0–13.0]	0.002 *

Data are reported as n, mean (standard deviation), or median [interquartile range]. BMI: body mass index. GLFS-5: The 5-question Geriatric Locomotive Function Scale. KCL: Kihon Checklist. LS: locomotive syndrome. * *p* < 0.05.

**Table 3:** Comparison of locomotive syndrome and frailty items between the happy and unhappy groups.

		Happy Group (n = 76)	Unhappy Group (n = 35)	*p* Value
Sex, female		37, 48.7%	13, 37.1%	0.256
Height, cm		157.7 (9.3)	159.2 (10.2)	0.421
Weight, kg		57.8 (10.6)	58.4 (10.2)	0.780
BMI, kg/m^2^		23.1 (2.9)	23.0 (3.1)	0.794
Living alone		5, 6.6%	4, 11.4%	0.459
Engaging in hobbies		44, 57.9%	12, 34.3%	0.021 *
Community activity participation		36, 47.4%	15, 42.9%	0.658
Hypertension		47, 61.8%	21, 60.0%	0.853
Hyperlipidemia		8, 10.5%	7, 20.0%	0.232
Cancer		6, 7.9%	2, 5.7%	1.000
LS				
Prevalence		17, 22.4%	15, 42.9%	0.027 *
Q1 Going up and down stairs		0.0 [0.0–1.0]	1.0 [0.0–2.0]	0.022 *
Q2 Walking briskly		1.0 [0.0–1.0]	1.0 [0.0–2.0]	0.016 *
Q3 Keep waking without rest		1.0 [0.0–1.0]	1.0 [0.0–2.0]	0.196
Q4 Carrying objects 2 kg		0.0 [0.0–0.0]	1.0 [0.0–1.0]	0.003 *
Q5 Having load-bearing tasks		0.0 [0.0–1.0]	1.0 [0.0–1.0]	0.013 *
GLFS-5 total score		2.0 [0.0–5.0]	4.0 [1.0–7.5]	0.012 *
Frailty				
Status	Robust	40, 52.6%	9, 25.7%	0.011 *
	Pre-frailty	24, 31.6%	13, 37.1%	
	Frailty	12, 15.8%	13, 37.1%	
Domains	Activities of daily living	0.0 [0.0–1.0]	0.0 [0.0–1.0]	0.215
	Physical function	1.0 [0.0–2.0]	2.0 [0.0–2.0]	0.063
	Nutrition	0.0 [0.0–1.0]	0.0 [0.0–1.0]	0.956
	Oral function	0.0 [0.0–1.0]	1.0 [0.0–2.0]	0.031 *
	Outdoor activity	0.0 [0.0–0.0]	0.0 [0.0–1.0]	0.034 *
	Cognitive function	0.0 [0.0–0.0]	0.0 [0.0–1.0]	0.017 *
	Depression	0.0 [0.0–2.0]	2.0 [0.0–4.0]	0.001 *
KCL total score		3.0 [1.0–6.0]	6.0 [3.0–10.0]	0.002 *

Data are reported as n, mean (standard deviation), or median [interquartile range]. BMI: body mass index. GLFS-5: The 5-question Geriatric Locomotive Function Scale. KCL: Kihon Checklist. LS: locomotive syndrome. * *p* < 0.05.

**Table 4:** Comparison of locomotive syndrome and frailty items across the three subjective well-being groups, defined by a combination of life satisfaction and happiness.

		Satisfied–Happy (n = 76): A	Satisfied– Unhappy (n = 24): B	Unsatisfied–Unhappy (n = 11): C	*p* Value	Multiple Comparisons
Sex, female		37, 48.7%	9, 37.5%	4, 36.4%	0.545	
Height, cm		157.7 (9.3)	158.6 (10.9)	160.8 (8.8)	0.592	
Weight, kg		57.8 (10.6)	58.5 (11.3)	58.0 (7.6)	0.951	
BMI, kg/m^2^		23.1 (2.9)	23.2 (3.5)	22.4 (1.9)	0.705	
Living alone		5, 6.6%	2, 8.3%	2, 18.2%	0.319	
Engaging in hobbies		44, 57.9%	8, 33.3%	4, 36.4%	0.068	
Community activity participation		36, 47.4%	11, 45.8%	4, 36.4%	0.791	
Hypertension		47, 61.8%	13, 54.2%	8, 72.7%	0.644	
Hyperlipidemia		8, 10.5%	3, 12.5%	4, 36.4%	0.071	
Cancer		6, 7.9%	2, 8.3%	0, 0.0%	1.000	
LS						
Prevalence		17, 22.4%	7, 29.2%	8, 72.7%	0.004 *	^†^
Q1 Going up and down stairs		0.0 [0.0–1.0]	1.0 [0.0–1.0]	2.0 [1.0–2.0]	0.005 *	A < C
Q2 Walking briskly		1.0 [0.0–1.0]	1.0 [0.0–1.8]	2.0 [1.0–2.0]	0.004 *	A < C
Q3 Keep waking without rest		1.0 [0.0–1.0]	0.4 [0.0–1.8]	1.0 [1.0–3.0]	0.027 *	A < C
Q4 Carrying objects 2 kg		0.0 [0.0–0.0]	0.0 [0.0–1.0]	1.0 [0.0–1.0]	0.004 *	A < C
Q5 Having load-bearing tasks		0.0 [0.0–1.0]	0.0 [0.0–1.0]	1.0 [1.0–2.0]	0.008 *	A < C
GLFS-5 total score		2.0 [0.0–5.0]	3.0 [1.0–6.8]	7.0 [4.0–9.0]	0.003 *	A < C
Frailty						
Status	Robust	40, 52.6%	8, 33.3%	1, 9.1%	0.018 *	^††^
	Pre-frailty	24, 31.6%	8, 33.3%	5, 45.5%		
	Frailty	12, 15.8%	8, 33.3%	5, 45.5%		
Domains	Activities of daily living	0.0 [0.0–1.0]	0.0 [0.0–1.0]	0.0 [0.0–1.0]	0.285	
	Physical function	1.0 [0.0–2.0]	2.0 [0.0–2.0]	2.0 [1.0–3.0]	0.066	
	Nutrition	0.0 [0.0–1.0]	0.0 [0.0–1.0]	0.0 [0.0–1.0]	0.943	
	Oral function	0.0 [0.0–1.0]	1.0 [0.0–1.0]	2.0 [0.0–3.0]	0.017 *	A < C
	Outdoor activity	0.0 [0.0–0.0]	0.0 [0.0–0.8]	0.0 [0.0–1.0]	0.049 *	
	Cognitive function	0.0 [0.0–0.0]	0.0 [0.0–1.0]	1.0 [0.0–1.0]	0.039 *	
	Depression	0.0 [0.0–2.0]	1.5 [0.0–3.0]	4.0 [1.0–5.0]	<0.001 *	A < C
KCL total score		3.0 [1.0–6.0]	6.0 [1.0–8.8]	7.0 [5.0–13.0]	0.002 *	A < C

Data are reported as n, mean (standard deviation), or median [interquartile range]. BMI: body mass index. GLFS-5: The 5-question Geriatric Locomotive Function Scale. KCL: Kihon Checklist. LS: locomotive syndrome. * *p* < 0.05. ^†^ In residual analysis, non-LS in the Satisfied–Happy group was significantly higher than expected, while LS in the Unsatisfied–Unhappy group was significantly higher than expected. ^††^ In residual analysis, robust status in the Satisfied–Happy group was significantly higher than expected, while frailty in the Satisfied–Happy group and robust status in the Unsatisfied–Unhappy group were significantly lower than expected.

**Table 5:** Association of locomotive syndrome with life satisfaction and happiness: Binary logistic regression analysis.

	Crude Model				Model I			
	OR	95% CI		*p* Value	OR	95% CI		*p* Value
		Min	Max			Min	Max	
Satisfied–Happy	Ref				Ref			
Satisfied–Unhappy	1.4	0.5	4.0	0.498	1.6	0.6	4.5	0.391
Unsatisfied–Unhappy	9.3	2.2	38.8	0.002 *	10.5	2.4	45.5	0.002 *

CI: confidence interval, Max: maximum, Min: minimum, OR: odds ratio. * *p* < 0.05. Dependent variable: locomotive syndrome = 1, non-locomotive syndrome = 0. Model I: Includes adjustment for sex and body mass index. Unsatisfied–Happy: No participants.

**Table 6:** Association of frailty with life satisfaction and happiness: Binary logistic regression analysis.

	Crude Model				Model I			
	OR	95% CI		*p* Value	OR	95% CI		*p* Value
		Min	Max			Min	Max	
Satisfied–Happy	Ref				Ref			
Satisfied–Unhappy	2.7	0.9	7.6	0.067	3.0	1.0	8.9	0.052
Unsatisfied–Unhappy	4.4	1.2	16.9	0.029 *	4.5	1.1	17.8	0.031 *

CI: confidence interval, Max: maximum, Min: minimum, OR: odds ratio. * *p* < 0.05. Dependent variable: frailty = 1, pre-frailty or robust = 0. Model I: Includes adjustment for sex and body mass index. Unsatisfied–Happy: No participants.

## Data Availability

The data that support the findings of this study are not publicly available due to privacy restrictions and agreements with the municipal government and are therefore not available for sharing.

## Abbreviations

The following abbreviations are used in this manuscript:

BMI	body mass index
CI	confidence interval
GLFS-25	25-question Geriatric Locomotive Function Scale
GLFS-5	5-question Geriatric Locomotive Function Scale
KCL	Kihon Checklist
LS	locomotive syndrome
